# Prediction of ventricular arrhythmias and sudden cardiac death by quantification and location of late gadolinium enhancement on cardiac magnetic resonance: a systematic review and meta-analysis

**DOI:** 10.1093/europace/euaf214

**Published:** 2025-11-07

**Authors:** Thomas Nia Jensen, Sharif Omara, Jens Cosedis Nielsen, Michelle Samuel, Rob J van der Geest, Won Yong Kim, Katja Zeppenfeld

**Affiliations:** Department of Cardiology and Aarhus University, Department of Clinical Medicine, Aarhus University Hospital, 8200, Aarhus N, Denmark; Department of Cardiology, Leiden University Medical Center, P.O. Box 9600, 2300 RC, Leiden, Netherlands; Department of Cardiology and Aarhus University, Department of Clinical Medicine, Aarhus University Hospital, 8200, Aarhus N, Denmark; Willem Einthoven Centre for Cardiac Arrhythmia Research and Management (WECAM), 2300 RC, Leiden, Netherlands; Willem Einthoven Centre for Cardiac Arrhythmia Research and Management (WECAM), 8200, Aarhus N, Denmark; Mario Lemieux Center for Heart Rhythm Care, Allegheny Health Network and Drexel University, Pittsburgh, USA; Dalhousie University, B3H 4R2, Halifax, Canada; Department of Radiology, Leiden University Medical Center, 2300 RC, Leiden, Netherlands; Department of Cardiology and Aarhus University, Department of Clinical Medicine, Aarhus University Hospital, 8200, Aarhus N, Denmark; Department of Cardiology and Aarhus University, Department of Clinical Medicine, Aarhus University Hospital, 8200, Aarhus N, Denmark; Department of Cardiology, Leiden University Medical Center, P.O. Box 9600, 2300 RC, Leiden, Netherlands; Willem Einthoven Centre for Cardiac Arrhythmia Research and Management (WECAM), 2300 RC, Leiden, Netherlands; Willem Einthoven Centre for Cardiac Arrhythmia Research and Management (WECAM), 8200, Aarhus N, Denmark

**Keywords:** Ventricular tachycardia, Late gadolinium enhancement, Magnetic resonance imaging, Risk prediction, Meta-analysis

## Abstract

**Aims:**

In non-ischaemic cardiomyopathy (NICM), late gadolinium enhancement (LGE) detected by cardiovascular magnetic resonance is related to ventricular arrhythmia (VA) and sudden cardiac death (SCD) risk. The incremental prognostic value of quantifying LGE volume or mass beyond its mere presence, however, remains unresolved. The aim was to evaluate whether LGE quantification improves the prediction of SCD.

**Methods and results:**

PubMed, Embase, and Web of Science were searched on 20 November 2024 for observational studies that related quantified LGE burden to ventricular arrhythmia (VA)/SCD in NICM. Forty-one studies met prespecified criteria. Hazard ratios (HRs) were pooled with random-effects models, and quantification information was depicted in figures. Presence of any LGE was associated with a three-fold increase in VA/SCD risk (pooled HR 3.31, 95% confidence interval: 2.58–4.24). Beyond this binary marker, every additional 1% (or 1 g) of LGE was associated with a 12% relative risk increase (range 10–20%), independent of left ventricular ejection fraction and consistent across eight semi-automated thresholding techniques. This included 2–6 standard deviations above the reference myocardium and the full-width half-maximum method. Additionally, results were prone to substantial methodological heterogeneity (*τ*² = 1.49) and small-study bias. Once the presence of LGE was accounted for, scar quantification and location conferred minimal additional prognostic value.

**Conclusion:**

Quantitative LGE assessment provides little incremental prognostic utility over dichotomous LGE detection. Consensus imaging standards and prospective validation are requisite before LGE burden can guide primary implantable cardioverter defibrillator allocation in NICM.

## Graphical Abstract

**Graphical Abstract:**
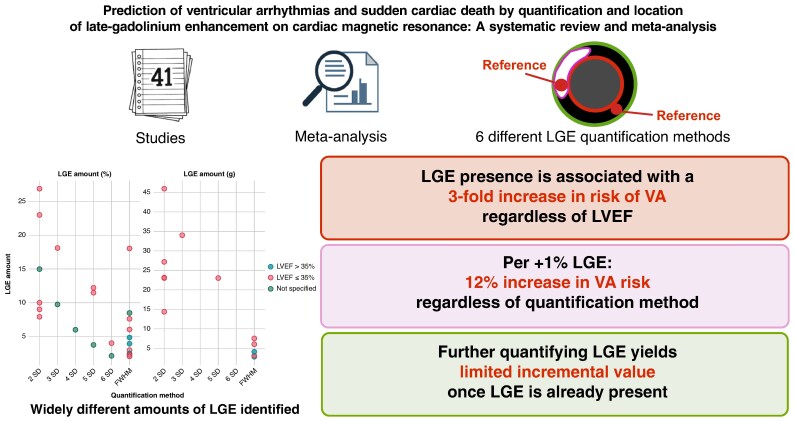

What’s new?

- There is a remarkable diversity in late gadolinium enhancement (LGE) quantification methods across studies.
- A dichotomous assessment of LGE (presence vs. absence) captures the majority of ventricular arrhythmic (VA) risk, while quantitative LGE analysis provides limited additional prognostic value.
- Septal, mid-wall, and subepicardial scars convey similar risks of VA and do not refine prediction further
- Based on the available data, no general applicable LGE threshold for identifying a patient at risk of VA can be identified.

## Introduction

Non-ischaemic cardiomyopathy (NICM) is an umbrella term comprised of different diseases with different underlying aetiologies spanning gene variants, toxins, autoimmunity, infections, storage diseases, and tachyarrythmias.^1^ A third of patients with NICM die suddenly due to ventricular arrhythmia (VA).^2^ Primary prevention of sudden cardiac death (SCD) is commonly achieved using implantable cardioverter defibrillators (ICD), a Class IIa recommendation for NICM patients with symptomatic heart failure and a left ventricular ejection fraction (LVEF) of ≤35%.^3,4^ This recommendation is extrapolated from studies including both ischaemic cardiomyopathy and NICM. However, there is increasing evidence that LVEF is a poor predictor of VA in patients with NICM.^5–8^

Depending on the characteristics, non-ischaemic myocardial fibrosis can create areas of delayed or blocked conduction, ultimately resulting in substrates for VA.^9–12^ Late gadolinium enhancement (LGE) cardiac magnetic resonance imaging (CMR) is a recognized tool for identifying localized myocardial fibrosis. However, its predictive value for VA remains a subject of ongoing debate.^1,2^

Studies have sought to define thresholds for the amount of LGE that warrant ICD implantation ranging from 5 to 20% of LGE as a percentage of total myocardium.^13–15^ Different semi-automated methods to quantify LGE have been proposed that rely on signal intensity thresholds using either healthy remote myocardium and/or severely diseased myocardium as a reference. These methods lack consensus, and even smaller amounts of LGE may be associated with adverse outcomes, which may depend on location and underlying aetiology.^14,16–18^

This systematic review and meta-analysis aims to evaluate whether LGE quantification improves the prediction of SCD, to investigate the existence and determination of optimal and universally applicable LGE thresholds, and to assess the potential of incorporating specific LGE distribution patterns to enhance SCD risk stratification.

## Methods

We performed a systematic review and meta-analysis of observational studies describing associations between the presence, amount, and location of LGE and the incidence of VA in patients with NICM. Studies had to detail their CMR methods to be included. VA was defined as SCD, sustained ventricular tachycardia (VT), ventricular fibrillation (VF), aborted SCD, or appropriate ICD intervention. The total inclusion and exclusion criteria are described in the Appendix. The Preferred Reporting Items for Systematic reviews and Meta-Analyses (PRISMA) guidelines were followed.^19^ The review was performed using a preplanned protocol in November 2024.

The databases Pubmed, Embase, and Web of Science were searched using the search string: (‘late gadolinium’ OR ‘LGE’ OR ‘contrast enhancement’ OR ‘delayed enhancement’ OR ‘late enhancement’) AND (‘nonischemic’ OR ‘nonischaemic’ OR ‘non ischaemic’ OR ‘non ischaemic’ OR ‘dilated cardiomyopath*’ OR ‘DCM’) AND (‘arrhythm*’ OR ‘sudden cardiac death’ OR ‘SCD’ OR ‘SCA’ OR ‘cardiac arrest*’ OR ‘OHCA’ OR ‘ventricular tachycardia*’ OR ‘ventricular tachyarrhythmia*’ OR ‘nonsustained ventricular tachycardia*’ OR ‘ventricular fibrillation*’ OR ‘CRT-D’ OR ‘ICD’ OR ‘defibrillator*’). The search was performed on 20 November 2024. Identified studies were screened by two independent reviewers (T.N.J. and S.O.) first by title and abstract followed by full-text review using the Covidence software. Discrepancies between reviewers were resolved through consensus after re-evaluation.

Data extraction was performed manually by the same two independent reviewers. In case of discrepancies, these data were assessed by both reviewers to reach consensus. The information extracted included baseline information of the study cohorts, data on associations between presence, amount and location of LGE and VA, and any information on quantification of LGE from LGE-MRI. Additionally, the quality of each included article was assessed using the Newcastle–Ottawa scale by two independent reviewers.^20^ Discrepancies were evaluated by both reviewers to reach consensus.

### Statistical analysis

A forest plot was constructed to denote the association between the presence of any LGE and the risk of VA compared with the absence of LGE. The forest plot was also stratified according to LVEF ≤35% or >35%. Summaries were calculated based on a DerSimonian–Laird random-effects model and common effect using inverse variance weighting.

To describe the relation between the amount of LGE and the applied LGE quantification method, scatterplots were constructed for LGE volume in % and grams. Studies were grouped into either LVEF ≤35% or >35% according to the mean/median LVEF in the baseline characteristics of the studies (whichever was available). Additionally, the association between the quantification method and risk of VA per % and grams was depicted in a figure.

Publication bias was assessed using a funnel plot, which displayed hazard ratios (HRs) (log scale) against their standard errors. We evaluated funnel plot asymmetry using Egger’s linear regression test. For studies reporting both adjusted and unadjusted HRs, adjusted values were used. Between-study heterogeneity was quantified using the *I*^2^ categorized as low (≤25%), moderate (26–50%), and high (>50%) heterogeneity.

Lastly, forest plots including studies that described the association between VA and specific locations of LGE enhancement were constructed. Estimates that described patchy/diffuse distributions or a combination of locations were not included in the analyses. Summary statistic within each group was calculated based on the DerSimonian–Laird random-effects model and the common effect using inverse variance weighting.

Synthesis of summary information and construction of figures were performed using R version 4.0.4 (2021-02-15).

## Results

**Figure 1:**
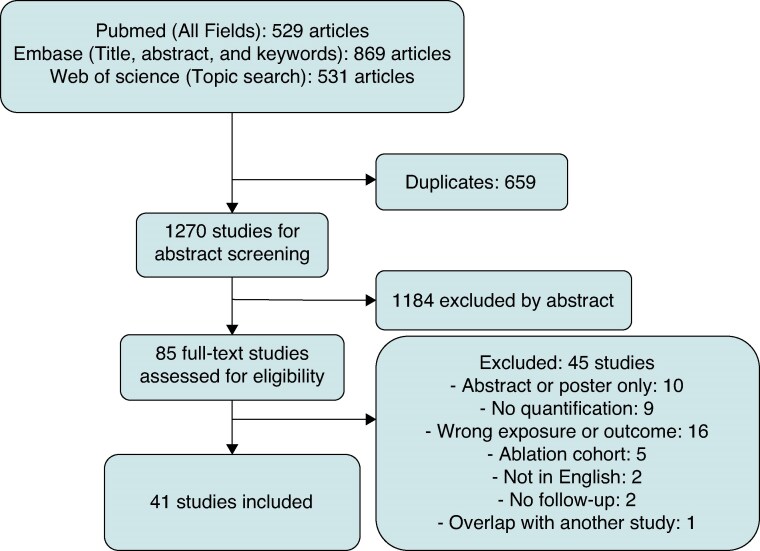
Study selection.

Article selection is described in *Figure 1*. The search yielded 2029 unique studies. Out of these, 1270 were screened by title and abstract studies, 85 were full-text screened, and 41 met the inclusion criteria. A list of all included studies can be found in the appendix.

### Baseline characteristics

*Table 1* shows the baseline characteristics of the included studies.^10,13–18,21–54^ The total number of patients across all studies was 14 658 with a median of 179 patients (IQR: 115–509) per study. A majority of studies based their inclusion criteria on the WHO (32%) or European Society of Cardiology guidelines (20%) from when the study was performed, or the absence of ischaemic heart disease (38%). On average, 69.2% [standard deviation (SD): 9.9] of patients were male and 54.5 (SD: 6.2) years of age. Out of the 41 studies, 12% defined their inclusion criteria as LVEF <50%, 61% defined no limit, and 27% reported ‘reduced’ or ‘systolic dysfunction’ with no reference to a specific LVEF as their inclusion criteria. Although LVEF inclusion criteria differed in terms of LVEF, the actually recorded mean/median LVEF were markedly similar. Studies that described LVEF <50% in their inclusion criteria averaged 29.4 (SD: 4.6) in LVEF; studies with ‘reduced’ inclusion criteria, 34.7 (SD: 5.8); and all patients, 34.7 (SD: 11.1). Assessment of included studies using the Newcastle–Ottawa scale demonstrated that the majority (*n* = 32,  78 %) received a Newcastle–Ottawa score of 7–9 (‘high quality’). The remaining nine studies (22 %) scored 4–6 (‘moderate quality’). No study was categorized as ‘low quality’ (0–3 points) (see Supplementary material online, *Table S1*).

**Table 1:** Baseline information

Study	Non-ischaemic DCM criteria	Patients	Age	Male	LVEF at inclusion
LVEF at inclusion ≤35%					
Becker 2023^21^	ESC criteria	509	60 (±13)	57	28 (IQR: 20–38)
Claver 2022^22^	Absence of IHD	1165	60 (IQR: 50–69)	76	35 (IQR: 26–44)
Mirelis 2022^23^	ESC criteria	600	53 (±14)	66	34 (±11)
Piers 2022^24^	Not specified	115	59 (±12)	77	33 (±13)
Chen 2021^25^	ESC criteria	157	52 (±16)	71	27 (±11)
Infante 2021^26^	Absence of IHD	86	45 (±16)	80	30 (±13)
Alba 2020^27^	Idiopathic DCM	1672	57 (IQR: 45–66)	71	33 (IQR: 24–43)
Barison 2020^16^	ESC criteria	183	66 (IQR: 56–73)	73	24 (IQR: 21–31)
Behera 2020^28^	WHO criteria	112	40 (IQR: 25–55)	68	33 (IQR: 27–41)
Elming 2020^29^	Absence of IHD	236	61 (IQR: 54–68)	74	35 (IQR: 27–45)
Li 2020^30^	Absence of IHD, ≤ 60 years	395	40 (±14)	80	29 (±6)
Muthalaly 2019^31^	ESC criteria	130	55 (±15)	83	29 (±14)
Park 2018^32^	WHO criteria	378	54 (±14)	62	26.3 (±11)
Chimura 2017**^33^**	WHO criteria	179	61 (±15)	68	33 (±9)
Mikami 2016^14^	Absence of IHD	118	57 (±14)	58	32 (±12)
Shin 2016^34^	WHO criteria	365	54 (±15)	62	27 (±11)
Chen 2015^35^	Absence of IHD	59	58 (±18)	80	35 (±18)
Chimura 2015^36^	WHO criteria	175	60 (±15)	63	29 (±5)
Piers 2015^37^	WHO criteria	87	56 (±13)	62	29 (±12)
Perazzolo Marra 2014^38^	WHO criteria	137	59 (IQR: 43–70)	79	30 (IQR: 29–40)
Yamada 2014^39^	Absence of IHD	57	55 (±13)	70	33 (±12)
Neilan 2013^15^	WHO criteria	162	55 (±14)	65	26 (±8)
Gao 2012^13^	Absence of IHD	65	61 (±11)	81	26 (±7)
Leyva 2012^40^	Absence of IHD	97	64 (±10)	60	16 (±6)
Wu 2008^41^	Absence of IHD	32	55 (±11)	65	24 (±9)
LVEF at inclusion >35% and <60%					
Castrichini 2024^42^	ESC criteria	462	43 (±15)	58	44 (±14)
Hammersley EHJHF 2024^43^	CMR	355	54 (IQR: 43–64)	60.8	49 (IQR: 46–54)
Hammersley JACC 2024^44^	ESC criteria	866	53 (±15)	65	42 (±14)
Li 2023^45^	WHO criteria	466	44 (±14)	77	40 (±3)
Balaban 2022^46^	Absence of IHD	156	58 (±19)	82	38 (±18)
DeAngelis 2022^47^	ESC criteria	611	53 (±14)	71	37 (±10)
DiMarco 2022^48^	Absence of IHD	703	59 (IQR: 49–68)	66	42 (IQR: 32–48)
Purmah 2022^18^	Absence of IHD	719	57 (IQR: 47–65)	72	40 (IQR: 29–47)
Balaban 2021^49^	WHO criteria	156	56 (±14.5)	82	37 (±12)
Nakamori 2020^50^	Absence of IHD	115	54 (±13)	77	41 (±13)
Halliday 2019^17^	WHO criteria	874	52 (±15)	67	39 (±12)
Claridge 2017^51^	Absence of IHD	58	59 (±16)	79	–
Gulati 2013^10^	WHO criteria	472	51	69	37 (±13)
Assomull 2006^52^	WHO criteria	101	51 (±13)	69	36 (±12)
LVEF at inclusion ≥60%					
Gil 2023^53^	Absence of IHD and ICD	525	43 (±14)	30	63 (±6)
Lota 2021^54^	Mid-wall or subepicardial LV fibrosis	748	50 (IQR: 38–61)	62	66 (IQR: 62–70)

CMR, cardiac magnetic resonance; ESC, European Society of Cardiology; ICD, implantable cardioverter defibrillator; IHD, ischaemic heart disease; DCM, dilated cardiomyopathy; LV, left ventricular; LVEF, left ventricular ejection fraction; WHO, World Health Organization.

### Follow-up

Follow-up characteristics are shown in *Table 2*. The median follow-up time of all studies was 3.0 years (IQR: 2.3–4.9). The primary outcomes were predominantly composite endpoints. Out of the 41 studies included, 14 studies (34 %) used ICD shocks only; 17 (41 %) used ICD shock or antitachycardia pacing (ATP); 4 (10 %) did not specify whether ATP was included; and 6 (15 %) did not use ICD intervention in their outcome definition. Definitions varied slightly between studies but generally captured severe VA events. The most common outcome types were SCD (73%), appropriate ICD intervention (68%), VT (66%), VF (39%), and aborted SCD (39%). The median number of events was 25 events per 100 person-years (IQR: 13.5–45.5). The rate of events differed significantly between patients with LGE (median 14.8 events per 100 person-years (IQR: 9.0–19.4) and patients without LGE [median 2.9 events per 100 person-years (IQR: 0.8–5.6)] (*P* < 0.001).

**Table 2:** Follow-up information

Study	Outcome type	Follow time (median)	Total events	Events/100 person-years	Event rate (LGE)	Event rate (no LGE/below cut-off)
Castrichini 2024^42^	SCD; sustained VT or VF; appropriate ICD shock or ATP	81 months	98	3.1	3.1	1.3
Hammersley EHJHF 2024^43^	SCD; VT; VF; appropriate ICD shock	7.8 years	19	0.7	0.7	0.3
Hammersley JACC 2024^44^	SCD; VF; VT; appropriate ICD shock	7.6 years	52	0.8	–	–
Becker 2023^21^	SCD; VT; VF; appropriate ICD shock or ATP	2.7 years	29	2.1	–	–
Gil 2023^53^	VT; VF; VT ablation; appropriate ICD intervention (not specified)	5.8 years	27	0.9	–	–
Li 2023^45^	SCD	79 months	40	1.3	–	–
Balaban 2022^46^	SCD; VT; aborted SCD (not specified)	7.7 years	25	2.1	–	–
Claver 2022^22^	SCD; VT; appropriate ICD intervention (not specified)	36 months	74	2.1	13.6	1.3
DeAngelis 2022^47^	SCD; VT; VF; appropriate ICD intervention (not specified)	47 months	46	1.9	18.1	2.4
DiMarco 2022^48^	SCD; VT; appropriate ICD shock or ATP	21 months	14	1.1	–	–
Mirelis 2022^23^	SCD; VT; appropriate ICD shock	2.7 years	48	3	29	19
Piers 2022^24^	VT; appropriate ICD shock or ATP	24 h	43	13657.2	57	47
Purmah 2022^18^	SCD; VT; appropriate ICD intervention (not specified)	1044 days	45	2.2	–	–
Balaban 2021^49^	SCD; VT; appropriate ICD shock	1611 days	16	2.3	–	–
Chen 2021^25^	SCD; VT; VF; appropriate ICD shock or ATP	13 months	31	18.2	24	6
Infante 2021^26^	appropriate ICD shocks	4.9 years	7	1.7	10.9	3.2
Lota 2021^54^	SCD; appropriate ICD shock	4.3 years	1	0	0.2	0
Alba 2020^27^	SCD; appropriate ICD shock	2.3 years	88	2.3	12.1	5
Barison 2020^16^	Appropriate ICD shock	30 months	20	4.4	–	–
Behera 2020^28^	SCD; VT; appropriate ICD shock	745 days	11	4.8	20.4	2.9
Elming 2020^29^	SCD; VT; appropriate ICD shock	5.3 years	40	3.2	23.9	10.6
Li 2020^30^	SCD; appropriate ICD shock	3 years	93	7.8	–	–
Nakamori 2020^50^	SCD; VT; VF; appropriate ICD shock or ATP	24 months	13	5.7	–	–
Halliday 2019^17^	SCD; appropriate ICD shock	4.9 years	84	2	18.3	5.1
Muthalaly 2019^31^	SCD; VT; appropriate ICD shock or ATP	3.2 years	18	4.3	–	–
Park 2018^32^	SCD; VT; VF; appropriate ICD shock or ATP	44.3 months	–	–	–	–
Chimura 2017^33^	SCD; VT; VF	3.8 years	13	1.9	13	0
Claridge 2017^51^	Appropriate ICD shock or ATP	938 days	18	12.1	–	–
Mikami 2016^14^	SCD; appropriate ICD shock or ATP	1.9 years	15	6.7	16.1	2.7
Shin 2016^34^	SCD; VT; VF; appropriate ICD shock or ATP	44.3 months	44	3.3	–	–
Chen 2015^35^	VT; VF; appropriate ICD shock or ATP	425 days	–	–	–	–
Chimura 2015^36^	SCD; VT; VF; appropriate ICD shock or ATP	5.1 years	18	2	14.8	0
Piers 2015^37^	VT; VF; appropriate ICD shock or ATP	45 months	28	8.6	–	–
Perazzolo Marra 2014^38^	SCD; VT; VF; appropriate ICD shock or ATP	3 years	22	5.4	7	3.3
Yamada 2014^39^	VT; VF	71 months	1	0.3	4	0
Gulati 2013^10^	SCD; appropriate ICD shock	5.3 years	65	2.6	29.6	7
Neilan 2013^15^	SCD; appropriate ICD shock or ATP	29 months	37	9.5	–	–
Gao 2012^13^	SCD; appropriate ICD shock or ATP	632 days	8	7.1	15.2	5.3
Leyva 2012^40^	SCD	1038 days	3	1.1	15	0
Wu 2008^41^	Appropriate ICD shock	17 months	7	15.4	14.8	7.9
Assomull 2006^52^	SCD; VT	658 days	7	3.8	5	2

ATP, antitachycardia pacing; SCD, sudden cardiac death; LGE, late gadolinium enhancement; VT, ventricular tachycardia; VF, ventricular fibrillation; ICD, implantable cardioverter defibrillator.

### Presence or absence of LGE

**Figure 2:**
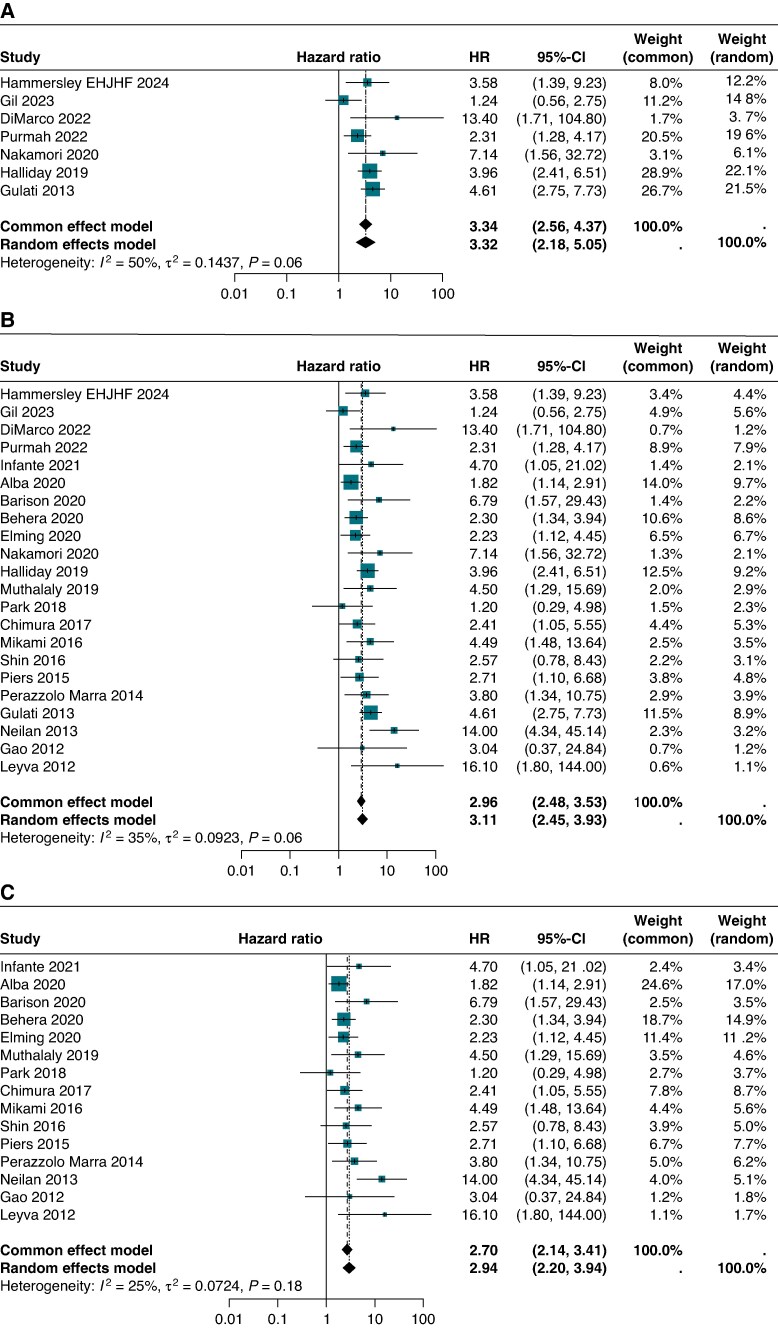
Any LGE. All studies. Studies with LVEF ≤35%. Studies with LVEF >35%. HR, hazard ratio; LVEF, left ventricular ejection fraction.

*Table 3* depicts the distribution of risk associated with VA for any LGE. All studies showed that any LGE was associated with an increased risk of VA with the majority being significant results. Pooled analysis (*Figure 2*) showed a more than three times increased risk of VA if any LGE was present {HR 3.11 [95% confidence interval (CI): 2.45–3.93]; *I*² = 35%}. Both pooled analyses of patients with LVEF ≤ 35% [HR 3.13 (95% CI: 2.25–4.36); *I*² = 25%] and LVEF > 35% [HR 3.12 (95% CI: 2.17–4.49); *I*² = 50%] showed similar results to the overall pooled analysis.

**Table 3:** Quantification

Study	LGE_volume_percent	LGE_volume_g	HR_detail	Quantified_HR_unadjusted	Quantified_HR_adjusted
2SD					
Hammersley JACC 2024^44^	–	–	Per 1g	1.05 (95% CI: 1.04–1.07)	1.05 (95% CI: 1.03–1.07)
Behera 2020^28^	–	–	>14% LV	6.18 (95% CI: 1.87–20.37)	8.89 (95% CI: 2.62–28.86)
Claridge 2017^51^	15 (±13.3)	–	Per 1%	1.04 (95% CI: 1.02–1.08)	1.04 (95% CI: 1–1.07)
Mikami 2016^14^	–	–	Per 1%	1.02 (95% CI: 1–1.05)	–
Mikami 2016^14^	–	–	Per 1% (Septal only)	1.11 (95% CI: 1.06–1.17)	–
Mikami 2016^14^	–	–	>10.15%:	6.09 (95% CI: 2.12–17.5)	–
Chen 2015^35^	23 (±8)	27.2 (±18.2)	Per 1%	1.09 (95% CI: 1.05–1.14)	1.1 (95% CI: 1.04–1.15)
Perazzolo Marra 2014^38^	–	–	Per 1%	1.04 (95% CI: 0.98–1.09)	–
Yamada 2014^39^	7.9 (IQR: 2.9–22)	23 (IQR: 8.4–64)	–	–	–
Neilan 2013^15^	9 (±5)	14.4 (±9.3)	Per 1%	1.17 (95% CI: 1.12–1.22)	–
Gao 2012^13^	26.9 (±24.4)	46 (±38)	Per 10g	1.31 (95% CI: 1.08–1.6)	–
Wu 2008^41^	10 (±13)	23.2 (±32.9)	–	–	–
Assomull 2006^52^	–	–	–	–	–
3SD					
Hammersley JACC 2024^44^	–	–	Per 1g	1.16 (95% CI: 1.11–1.22)	1.14 (95% CI: 1.08–1.2)
Claridge 2017^51^	9.7 (±9)	–	–	1.05 (95% CI: 1.01–1.08)	1.04 (95% CI: 0.99–1.08)
Mikami 2016^14^	–	–	Per 1%	1.02 (95% CI: 1–1.04)	–
Mikami 2016^14^	–	–	Septal LGE (per 1%)	1.12 (95% CI: 1.07–1.18)	–
Mikami 2016^14^	–	–	>6.63%:	5.68 (95% CI: 2.11–15.28)	–
Gao 2012^13^	18.1 (±20.7)	34 (±38)	Per 10g	1.34 (95% CI: 1.1–1.63)	–
4SD					
Claridge 2017^51^	6 (±5.8)	–	–	1.04 (95% CI: 0.99–1.09)	–
5SD					
Hammersley JACC 2024^44^	–	–	–	–	–
Chen 2021^25^	11.5 (IQR: 4.4–23)	–	–	1.04 (95% CI: 1.01–1.06)	1.04 (95% CI: 1.01–1.06)
Chen 2021^25^	–	–	–	–	1 (95% CI: 0.96–1.04)
Li 2020^30^	–	–	>14%:	4.82 (95% CI: 1.88–12.36)	–
Claridge 2017^51^	3.7 (±3.6)	–	–	1.04 (95% CI: 0.98–1.09)	–
Mikami 2016^14^	–	–	Per 1%	1.03 (95% CI: 0.99–1.06)	–
Mikami 2016^14^	–	–	Per 1% (Septal only)	1.21 (95% CI: 1.1–1.31)	–
Mikami 2016^14^	–	–	>2.74%:	8.65 (95% CI: 3.06–24.51)	–
Gao 2012^13^	12.2 (±18.4)	23 (±34)	Per 10g	1.39 (95% CI: 1.11–1.74)	–
6SD					
Barison 2020^16^	4 (IQR: 2–11)	–	Percent LGE mass	2.32 (95% CI: 1.44–3.75)	–
Claridge 2017^51^	2.1 (±2.4)	–	–	1.01 (95% CI: 0.97–1.04)	–
FWHM					
Hammersley JACC 2024^44^	–	–	–	–	–
Li 2023^45^	3.9 (±5.2)	4.1 (±5.7)	>7.1%	4.9 (95% CI: 2.6–9.1)	4.4 (95% CI: 2.4–8.3)
Piers 2022^24^	–	6 (IQR: 2–11)	–	2.09 (95% CI: 0.9–4.85)	1.23 (95% CI: 0.5–3.07)
Balaban 2022^46^	–	–	Per quartile	1.3 (95% CI: 0.9–1.8)	–
DeAngelis 2022^47^	4.8 (IQR: 2.3–14.2)	–	Includes ICM	1.02 (95% CI: 1–1.04)	–
Mirelis 2022^23^	2.3 (±6.8)	3 (±8.7)	–	–	–
Balaban 2021^49^	–	–	Volume	1.37 (95% CI: 0.96–1.95)	1.44 (95% CI: 1.07–1.94)
Balaban 2021^49^	–	–	Interface area	1.55 (95% CI: 1.06–2.28)	1.75 (95% CI: 1.24–2.47)
Lota 2021^54^	2.2 (IQR: 1.2–4.1)	2.8 (IQR: 1.5–5.2)	–	–	–
Elming 2020^29^	3 (IQR: 1.4–7.1)	7.5 (IQR: 3.1–13.7)	Per 1%	1.07 (95% CI: 1.01–1.14)	–
Li 2020^30^	–	–	>14%	3.64 (95% CI: 1.66–8)	3.33 (95% CI: 1.48–7.5)
Halliday 2019^17^	–	–	–	–	2.79 (95% CI: 1.42–5.49)
Halliday 2019^17^	–	–	–	–	3.86 (95% CI: 2.09–7.13)
Halliday 2019^17^	–	–	–	–	4.87 (95% CI: 2.78–8.53)
Muthalaly 2019^31^	2 (±6)	–	–	1.1 (95% CI: 1.06–1.17)	–
Park 2018^32^	7.6 (±12.1)	–	–	–	–
Claridge 2017^51^	8.4 (±8.6)	–	–	1.05 (95% CI: 1.01–1.09)	1.04 (95% CI: 0.99–1.09)
Shin 2016^34^	–	–	<8%	2.54 (95% CI: 0.85–7.6)	1.06 (95% CI: 0.32–3.53)
Shin 2016^34^	–	–	≥8%	8.23 (95% CI: 2.84–23.8)	2.57 (95% CI: 0.78–8.4)
Piers 2015^37^	–	2.8 (IQR: 0–5.8)	Per 10g	1.47 (95% CI: 1.1–1.97)	–
Chen 2015^35^	18 (±8)	–	–	–	–
Gulati 2013^10^	2.5 (IQR: 1.2–4.8)	–	–	–	1.1 (95% CI: 1.05–1.16)
Neilan 2013^15^	6 (±4)	–	–	–	–
MAD SD					
Nakamori 2020^50^	3.2 (IQR: 2.1–6.2)	–	Per 1%	1.29 (95% CI: 1.06–1.56)	–

CI, confidence interval; IQR, interquartile range; HR, hazard ratio.

### Methods of quantification of LGE

**Figure 3:**
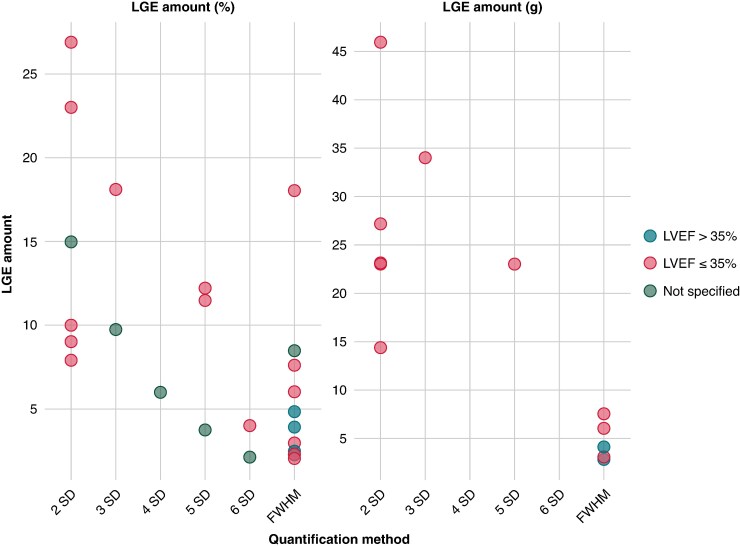
LGE amount based on the LGE quantification method. FWHM, full width at half maximum; LGE, late gadolinium enhancement; LVEF, left ventricular ejection fraction; SD, standard deviation.

The LGE quantification methods applied in the included studies can be found in *Table 3*. The identified methods encompassed either an SD-based thresholding or the full-width half-maximum (FWHM) method. Only one study employed a modified SD method known as mean absolute deviation (MAD) SD and was excluded from the comparison in *Figure 3*. For SD-based thresholding above the mean signal intensity in unaffected myocardium, 23.6% of studies used 2SD, 10.9% used 3SD, one study used 4SD, 16.4% used 5SD, and two studies used 6SD. The FWHM method, which uses the half point between the maximum and minimum signal intensity as the threshold, was used by 41.8% of studies. In two of these studies, the threshold was determined from 50% of maximum signal intensity without reference to FWHM, but because these methods are identical, they were classified in the FWHM category. Studies employing FWHM were generally more recent, with most published from 2019 onwards, though SD-based methods have continued to be used throughout the period, with studies from 2006 to 2024. Additionally, some more recent studies explored alternative quantification methods, including T1 native mapping and T1 post-contrast measurements. A thorough description of the quantification methods can be found in the supplement.

### Amount of LGE

Amount of LGE was provided as percentage of total LV volume or grams. The amount varied between studies and quantification methods (*Figure 3*), and the choice of quantification method significantly influenced the amount of LGE detected. Lower thresholds, such as 2SD, generally resulted in larger amounts of LGE. However, considerable variability was observed even among studies employing the same quantification method. For instance, using the 2SD threshold, reported median values for enhancement extent ranged from 7.9 to 26.9%, with a median of 12.5%. Some studies reported high standard deviations of patient enhancement extent over 20%. Studies using 3SD reported values from 9.7 to 18.1%, while 4SD showed lower amounts (median: 6%). 5SD threshold methods showed variable results (ranging from 3.7 to 12.2%), with some more recent studies reporting higher values ∼11%. Studies using 6SD reported lower values (2.1–4.0%). Studies that utilized the FWHM method also showed considerable variation, with values ranging from 2.0 to 18.0%, with a median of 3.9%. The single study using MAD SD reported values (3.2%, IQR: 2.1–6.2%) similar to those found with higher SD thresholds and FWHM. When reported in grams rather than percentages, similar patterns were observed, with 2SD methods reporting values from 23.2 to 46.0 g, 3SD a median of 34.0 g, 5SD 23.0 g, and FWHM methods reporting between 3.5 and 7.5 g.

### Amount of LGE and risk of VA

**Figure 4:**
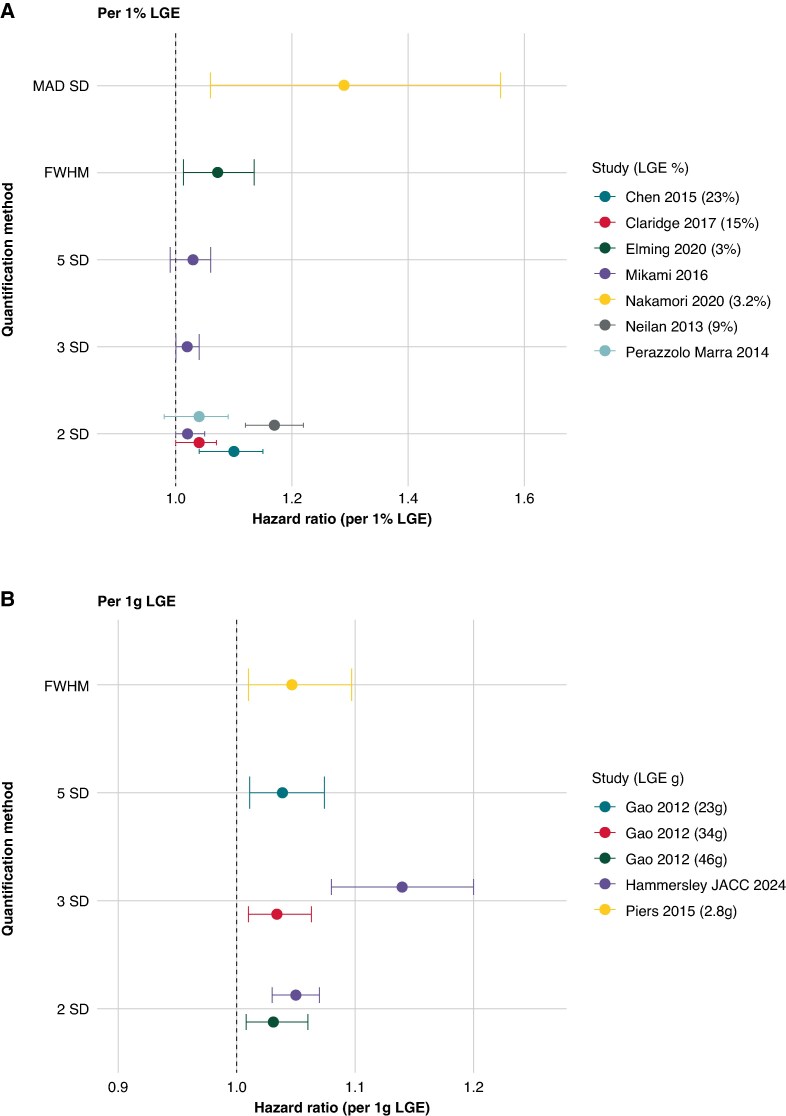
LGE amount and risk of a malignant arrhythmia. Studies without continuous measurement of outcomes were not included in the figure. Studies that provided grams/% as mean and standard deviation were converted to median and interquartile range, assuming a normal distribution of the cohort. LGE, late gadolinium enhancement.

After semi-automated quantification, all studies showed an increase in VA risk of approximately 10–20% per % or per gram LGE (*Figure 4*). Most studies that provided information on quantified risk utilized the 2SD method and provided per % estimates. The information gathered from *Figure 3* and *Figure 4* shows that despite largely varying amounts of LGE, most studies were similar in the per % or g increase in risk, regardless of the quantification method used or the amount of LGE identified in the cohort.

### Location

**Figure 5:**
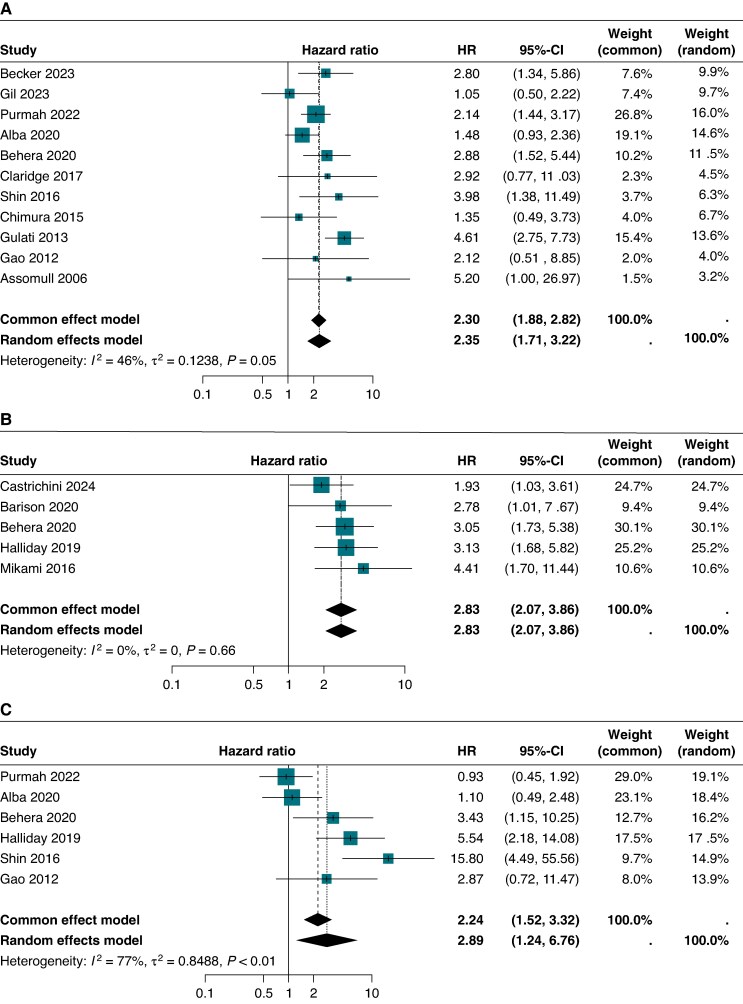
Locations: mid-wall, septal, and subepicardial. CI, confidence interval; HR, hazard ratio.

The location of LGE appeared to influence VA risk (*Figure 5*). The main locations explored were septal, mid-wall, and subepicardial, with some studies reporting combined patterns (septal + lateral mid-wall; mid-wall + subepicardial). On random-effects meta-analysis, septal LGE was associated with an HR of 2.83 (95% CI: 2.07–3.86), *I*² = 0%; mid-wall LGE with an HR of 2.35 (95% CI: 1.71–3.22), *I*² = 46%; and subepicardial LGE with an HR of 2.89 (95% CI: 1.24–6.76), *I*² = 77%.

### Publication bias

**Figure 6:**
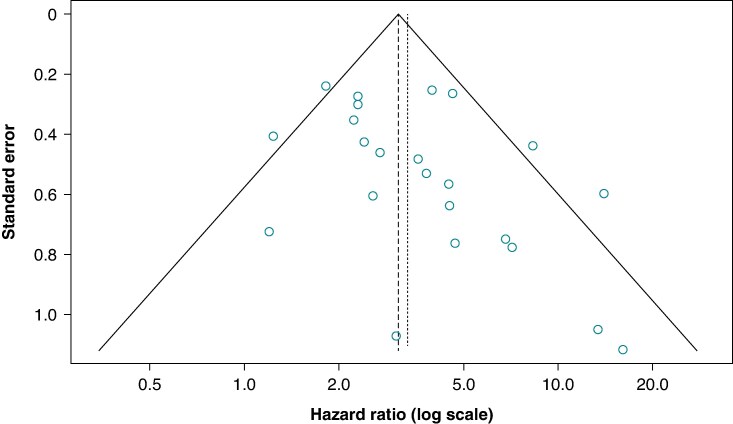
Funnel plot.

The funnel plot (*Figure 6*) showed an uneven pattern, with smaller studies unidirectionally reporting high HRs and high heterogeneity (*τ*² = 1.494). This pattern was supported by Egger’s test, which showed borderline significance (*P* = 0.056).

## Discussion

This systematic review is the first to evaluate the impact of both the extent and anatomical distribution of LGE on the prediction of VA, specifically within the context of studies employing semi-automated quantification techniques using varying thresholding methods to define LGE. We included 41 studies and over 12 000 patients with NICM who underwent LGE-CMR for VA risk stratification. The main findings can be summarized as follows: (i) any LGE is associated with an increased risk of VA; (ii) there is a remarkable diversity in LGE quantification methods across studies; (iii) the magnitude of VA risk does not vary by the amount of LGE detected across different methods; (iv) based on the available data, no general applicable LGE threshold for identifying a patient at risk can be identified; and (v) specific locations of LGE did not refine VA risk prediction.

### Any amount of LGE is associated with risk of VA

We found that the presence of any LGE was consistently associated with VA, regardless of LVEF. However, although most studies permitted the inclusion of patients with preserved LVEF, the majority ultimately included patients with comparably low LVEF, suggesting a potential patient inclusion bias. Additionally, current findings seem mainly valid for patients with reduced LVEF. Several studies showed wide confidence intervals, and the distribution of risk across studies is varied, although in all cases elevated. Publication bias may have contributed to this finding, as we observed a skewed distribution of smaller studies towards findings of significantly elevated risk. It is known that in early disease, the event rates are very low, and patients may be underrepresented.^55^ Nonetheless, when estimates from multiple studies are combined, we estimated that having any LGE is associated with a three-fold increase in VA. This finding was consistent among patients with mildly reduced LVEF and those with reduced LVEF. Previous meta-analyses, primarily focused on patients with reduced LVEF, have reported similar results.^10,27,52,56–61^ Pooling information from multiple studies provides a balanced approach by incorporating the moderate to high between-study heterogeneity, explained by both sampling error and genuine differences in study populations. Additionally, while our funnel plot suggests potential small-study effects, with smaller studies lightly biased towards estimates of significantly elevated risk, the meta-analysis adjusts for this discrepancy. This approach is particularly relevant for LGE studies in NICM, where varying protocols and patient characteristics may contribute to heterogeneous effect sizes.

### Diversity in LGE quantification methods

There was significant variability both between different quantification methods and among studies using the same methods, with some methods exceeding 20% in standard deviations in the percentage of LGE. Most studies provided LGE amount as a percentage of LV myocardium with a few describing it in grams. Naturally, LGE amount decreases with increasing SD thresholds. Of note, the FWHM method provided amounts similar to the 6SD threshold method, a similarity that has been shown previously also for post-MI scars and hypertrophic cardiomyopathies.^62^ However, NICM fibrosis is fundamentally different from post-MI scar.^63^ LGE can identify focal fibrosis but requires the contrast of normal surrounding myocardium and therefore cannot depict diffuse fibrosis. The FWHM technique has been reported to be the most reproducible but has an important limitation. It uses half the maximal signal of the brightest area assuming the presence of a confluent and compact scar as reference. However, non-ischaemic fibrosis is usually not compact, and the highest amount of regional fibrosis may vary across different aetiologies and individuals. The lack of a uniform reference may explain the range of reported amounts of LGE. Of note, the distinction between regional and diffuse interstitial fibrosis is not clear-cut, and the two phenomena can overlap to a variable extent in individual NICM patients.^64^ This has important implications for the SD methods that rely on an area of normal myocardium as reference, which may not exist in many patients with NICM. This is supported by the discrepancy between a relatively low volume or mass of LGE and the reduction in LVEF, supporting the coexistence of diffuse and localized fibrosis. The gold standard to validate the optimal quantification method would be the comparison with whole heart histology.

In the context of NICM, direct histopathological assessment of myocardial scar is limited, and most are based on endocardial biopsies, which may not represent the often inhomogeneous distribution of fibrosis.^52,63,65^

### Quantified LGE as it relates to risk of ventricular arrhythmia

An increase in risk of 10–20% per % or gram of LGE was observed. This was irrespective of the method used and total amount of LGE. Translating this stepwise risk increase into meaningful clinical decision-making may be difficult. While studies consistently show that increasing amounts of LGE are associated with higher risk, the amount of LGE identified in each patient is, as previously noted, largely determined by the quantification method used. NICM is an umbrella term comprised of multiple underlying aetiologies, fibrotic patterns, and risk profiles.^1^ Analysing these as a single unit may be less appropriate, as the different characteristics of fibrosis (diffuse vs. localized, septal vs. lateral) may be associated with different risks.^66^ Additionally, the architecture of fibrosis is an important determinant of arrhythmogenesis. Areas consisting of or containing longer strands of fibrosis had the highest propensity for conduction abnormalities related to VT.^67^ From a biological standpoint, a relationship between LGE amount and arrhythmic risk is plausible, but a linear relationship seems unlikely. Small increments in LGE may provide minimal value once LGE is already present. The architecture of fibrosis, whose delineation is currently beyond the resolution of LGE-CMR, and the location of LGE may contribute more to the clinical risk of the patient than the amount itself.^17,63,67^

### Identifying a threshold

An objective and universal threshold to identify LGE associated with arrhythmogenic substrate for VA may be unattainable within NICM. As discussed, signal intensity threshold is determined using a variation of methods, including semi-automatic quantification, which all rely also on observer input, thereby introducing further variability. Furthermore, it is potentially influenced by additional factors such as scanner field strength, contrast amount, and acquisition protocols.^32,35^ For instance, higher field strength scanners may offer improved contrast to noise, potentially enhancing LGE detection and delineation, but may also be associated with increased artefacts.^68,69^ Additionally, breathing artefacts in free-breathing protocols may blur pixels, leading to a washout effect that shifts colours towards grey rather than producing distinct enhancement. Currently, due to a lack of agreement on quantification protocols and acquisition, meaningful comparisons are currently limited to the same cohort, scanned on the same scanner, and using the same acquisition protocol. As a result, achieving external validation remains challenging. Even within our analyses on the presence of any LGE, between-study heterogeneity remains moderate to high and increases further with the addition of semi-automated quantification.

### Location of LGE to predict risk of VA

The location of LGE did not provide any meaningful stratification of high-risk patients as a singular factor. When all patients, regardless of aetiology, are combined in pooled estimates, the location of LGE did not seem to convey any additional information. Patients with fibrosis involving the interventricular septum and those with transmural fibrosis had a significantly higher incidence of VA, but patients with LGE in other regions were still at increased risk.^16,70^ While not meaningful as a singular factor, certain locations are often linked to specific genetic mutations, such as mid-wall fibrosis with Lamin A/C mutations.^14,16,36,71,72^ Some of these cardiomyopathies may be more arrhythmogenic, not solely attributable to the location of fibrosis or the amount of LGE. Combining LGE information with the underlying genetic profile may improve risk prediction in these patients.

### Future perspectives

To improve external validation, it remains important to work towards generally agreed acquisition and postprocessing protocols. Moreover, the impact of newer quantification methods, such as T1 mapping and extracellular volume measurements, remains to be fully examined.^48,50,73^ Combining genetic profiling with LGE may improve risk prediction.^23^ The impact of specific locations and patterns of LGE on arrhythmogenesis needs to be further explored, also considering the different underlying aetiologies.

## Limitations

Our systematic review and meta-analysis have several limitations. First, there was notable variation between studies, including differences in study groups, methods, outcomes, and follow-up periods. Although subgroup analyses were performed, some differences may still influence the overall results. Second, our funnel plot demonstrated risk of publication bias, as there were fewer studies with negative or unclear findings, possibly overstating the significantly elevated risk in patients with LGE. Third, the quality of included studies varied, which may have influenced our results. Fourth, most findings were based on observational studies, limiting the ability to conclude causation due to potential biases and confounding factors. Finally, results might not be widely generalizable due to differences in the populations studied, as well as differences in the protocols for obtaining LGE-MRI images. Many studies were conducted in specific groups or regions, which might not represent broader populations.

## Conclusions

In conclusion, LGE is consistently associated with VA across NICM, regardless of location and amount. Quantifying the location of LGE with any threshold does not appear to currently add significant value to the clinical interpretation of these findings. Standardized quantification methods, larger-scale studies with external validation, and further investigation into the prognostic value of specific LGE parameters are crucial for advancing this field and improving patient care. Furthermore, understanding how signal intensity is associated with arrhythmogenic substrate on a histological level seems crucial. Lastly, establishing a universal threshold for predicting VA seems unlikely due to the large variation in underlying aetiologies and arrhythmogenic properties of different NICMs.

## Supplementary Material

euaf214_Supplementary_Data

## Data Availability

No new data were generated in support of this research. All analysed data are part of previously published papers.
