# Supplementary material for: Prediction of ventricular arrhythmias and sudden cardiac death by quantification and location of late gadolinium enhancement on cardiac magnetic resonance: a systematic review and meta-analysis
Source: Europace. 2025 Nov 7;27(11):euaf214. doi: 10.1093/europace/euaf214 (PMC12598646; doi:10.1093/europace/euaf214)
Supplement: euaf214_Supplementary_Data [file euaf214_supplementary_data.docx]

**Quantification Manuscript - Appendix**

# Inclusion and exclusion criteria

*Inclusion criteria*

- Dilated cardiomyopathy
- E.g. muscular dystrophy-only cohort
- Alcohol
- Observational studies on association of LGE pattern and/or extent and VA
- Detailing CMR strategy (acquisition and post-processing)
- Arrhythmic endpoint (or combined but data available on arrhythmic endpoint separately)

*Exclusion criteria*

- Abstracts only: only published articles in peer-review journals
- NCCMP
- Sarcoidosis
- Post-myocarditis
- Exclusively right-sided cardiomyopathies
- Paediatric patients
- Athletes
- Ischemic cardiomyopathies
- VT ablation performed at baseline
- Inflammatory aetiologies for DCM

# Quantification methods

- **Standard Deviation (SD):** Involves calculating the mean signal intensity (SI) (the brightness of the pixel on the image) and its standard deviation (SD) within normal myocardium. LGE is defined in areas where the SI exceeds a threshold set at a certain number of SDs above the mean SI of the normal myocardium, commonly 2, 3, 5, or 6 SDs.
- **Full Width at Half Maximum (FWHM):** In LGE quantification, the full width at half maximum (FWHM) technique uses half the maximal signal intensity within the hyperenhanced region as the threshold.
- **Mean Absolute Deviation Standard Deviation (MAD SD):** Reduces the influence of outliers and noise by using the Mean Absolute Deviation (MAD) from the median SI within the normal myocardium instead of the standard deviation. The MAD is adjusted by a factor to approximate the standard deviation, which is then used to set the LGE threshold.

Included studies^1-41^

[1] Alba AC, Gaztanaga J, Foroutan F, Thavendiranathan P, Merlo M, Alonso-Rodriguez D, et al. Prognostic Value of Late Gadolinium Enhancement for the Prediction of Cardiovascular Outcomes in Dilated Cardiomyopathy: An International, Multi-Institutional Study of the MINICOR Group. *Circ Cardiovasc Imaging* 2020; **13**: e010105.

[2] Assomull RG, Prasad SK, Lyne J, Smith G, Burman ED, Khan M, et al. Cardiovascular magnetic resonance, fibrosis, and prognosis in dilated cardiomyopathy. *J Am Coll Cardiol* 2006; **48**: 1977-1985.

[3] Balaban G, Halliday BP, Hammersley D, Rinaldi CA, Prasad SK, Bishop MJ, et al. Left ventricular shape predicts arrhythmic risk in fibrotic dilated cardiomyopathy. *Europace* 2022; **24**: 1137-1147.

[4] Balaban G, Halliday BP, Porter B, Bai W, Nygaard S, Owen R, et al. Late-Gadolinium Enhancement Interface Area and Electrophysiological Simulations Predict Arrhythmic Events in Patients With Nonischemic Dilated Cardiomyopathy. *JACC Clin Electrophysiol* 2021; **7**: 238-249.

[5] Barison A, Aimo A, Mirizzi G, Castiglione V, Ripoli A, Panchetti L, et al. The extent and location of late gadolinium enhancement predict defibrillator shock and cardiac mortality in patients with non-ischaemic dilated cardiomyopathy. *Int J Cardiol* 2020; **307**: 180-186.

[6] Becker MAJ, van der Lingen ACJ, Cornel JH, van de Ven PM, van Rossum AC, Allaart CP, et al. Septal Midwall Late Gadolinium Enhancement in Ischemic Cardiomyopathy and Nonischemic Dilated Cardiomyopathy-Characteristics and Prognosis. *Am J Cardiol* 2023; **201**: 294-301.

[7] Behera DR, V KA, K KN, S S, Nair KKM, G S, et al. Prognostic value of late gadolinium enhancement in cardiac MRI of non-ischemic dilated cardiomyopathy patients. *Indian Heart J* 2020; **72**: 362-368.

[8] Castrichini M, De Luca A, De Angelis G, Neves R, Paldino A, Dal Ferro M, et al. Magnetic Resonance Imaging Characterization and Clinical Outcomes of Dilated and Arrhythmogenic Left Ventricular Cardiomyopathies. *J Am Coll Cardiol* 2024; **83**: 1841-1851.

[9] Chen W, Qian W, Zhang X, Li D, Qian Z, Xu H, et al. Ring-like late gadolinium enhancement for predicting ventricular tachyarrhythmias in non-ischaemic dilated cardiomyopathy. *Eur Heart J Cardiovasc Imaging* 2021; **22**: 1130-1138.

[10] Chen Z, Sohal M, Voigt T, Sammut E, Tobon-Gomez C, Child N, et al. Myocardial tissue characterization by cardiac magnetic resonance imaging using T1 mapping predicts ventricular arrhythmia in ischemic and non-ischemic cardiomyopathy patients with implantable cardioverter-defibrillators. *Heart Rhythm* 2015; **12**: 792-801.

[11] Chimura M, Kiuchi K, Okajima K, Shimane A, Sawada T, Onishi T, et al. Distribution of Ventricular Fibrosis Associated With Life-Threatening Ventricular Tachyarrhythmias in Patients With Nonischemic Dilated Cardiomyopathy. *J Cardiovasc Electrophysiol* 2015; **26**: 1239-1246.

[12] Chimura M, Onishi T, Tsukishiro Y, Sawada T, Kiuchi K, Shimane A, et al. Longitudinal strain combined with delayed-enhancement magnetic resonance improves risk stratification in patients with dilated cardiomyopathy. *Heart* 2017; **103**: 679-686.

[13] Claridge S, Mennuni S, Jackson T, Behar J, Porter B, Sieniewicz B, et al. Substrate-dependent risk stratification for implantable cardioverter defibrillator therapies using cardiac magnetic resonance imaging: The importance of T1 mapping in nonischemic patients. *Journal of Cardiovascular Electrophysiology* 2017; **28**: 785-795.

[14] Claver E, Di Marco A, Brown PF, Bradley J, Nucifora G, Ruiz-Majoral A, et al. Prognostic impact of late gadolinium enhancement at the right ventricular insertion points in non-ischaemic dilated cardiomyopathy. *Eur Heart J Cardiovasc Imaging* 2022: 8.

[15] De Angelis G, De Luca A, Merlo M, Nucifora G, Rossi M, Stolfo D, et al. Prevalence and prognostic significance of ischemic late gadolinium enhancement pattern in non-ischemic dilated cardiomyopathy. *Am Heart J* 2022; **246**: 117-124.

[16] Di Marco A, Brown PF, Bradley J, Nucifora G, Anguera I, Miller CA, et al. Extracellular volume fraction improves risk-stratification for ventricular arrhythmias and sudden death in non-ischaemic cardiomyopathy. *Eur Heart J Cardiovasc Imaging* 2022: 10.

[17] Elming MB, Hammer-Hansen S, Voges I, Nyktari E, Raja AA, Svendsen JH, et al. Myocardial fibrosis and the effect of primary prophylactic defibrillator implantation in patients with non-ischemic systolic heart failure-DANISH-MRI. *Am Heart J* 2020; **221**: 165-176.

[18] Gao P, Yee R, Gula L, Krahn AD, Skanes A, Leong-Sit P, et al. Prediction of arrhythmic events in ischemic and dilated cardiomyopathy patients referred for implantable cardiac defibrillator: evaluation of multiple scar quantification measures for late gadolinium enhancement magnetic resonance imaging. *Circ Cardiovasc Imaging* 2012; **5**: 448-456.

[19] Gil KE, Mikrut K, Mazur J, Black AL, Truong VT, Smart S, et al. Risk stratification in patients with structurally normal hearts: Does fibrosis type matter? *PLoS One* 2023; **18**: e0295519.

[20] Gulati A, Jabbour A, Ismail TF, Guha K, Khwaja J, Raza S, et al. Association of fibrosis with mortality and sudden cardiac death in patients with nonischemic dilated cardiomyopathy. *Jama* 2013; **309**: 896-908.

[21] Halliday BP, Baksi AJ, Gulati A, Ali A, Newsome S, Izgi C, et al. Outcome in Dilated Cardiomyopathy Related to the Extent, Location, and Pattern of Late Gadolinium Enhancement. *JACC Cardiovasc Imaging* 2019; **12**: 1645-1655.

[22] Hammersley DJ, Mukhopadhyay S, Chen X, Jones RE, Ragavan A, Javed S, et al. Precision prediction of heart failure events in patients with dilated cardiomyopathy and mildly reduced ejection fraction using multi-parametric cardiovascular magnetic resonance. *Eur J Heart Fail* 2024: 10.

[23] Hammersley DJ, Zegard A, Androulakis E, Jones RE, Okafor O, Hatipoglu S, et al. Arrhythmic Risk Stratification by Cardiovascular Magnetic Resonance Imaging in Patients With Nonischemic Cardiomyopathy. *J Am Coll Cardiol* 2024; **84**: 1407-1420.

[24] Infante AN, Koo CCY, Yip A, Lim YH, Yeo WT, Quek ST, et al. Magnetic resonance imaging of dilated cardiomyopathy: prognostic benefit of identifying late gadolinium enhancement in Asian patients. *Singapore Med J* 2021; **62**: 347-352.

[25] Leyva F, Taylor RJ, Foley PW, Umar F, Mulligan LJ, Patel K, et al. Left ventricular midwall fibrosis as a predictor of mortality and morbidity after cardiac resynchronization therapy in patients with nonischemic cardiomyopathy. *J Am Coll Cardiol* 2012; **60**: 1659-1667.

[26] Li S, Wang Y, Yang W, Zhou D, Zhuang B, Xu J, et al. Cardiac MRI Risk Stratification for Dilated Cardiomyopathy with Left Ventricular Ejection Fraction of 35% or Higher. *Radiology* 2023; **306**: e213059.

[27] Li X, Fan X, Li S, Sun W, Shivkumar K, Zhao S, et al. A Novel Risk Stratification Score for Sudden Cardiac Death Prediction in Middle-Aged, Nonischemic Dilated Cardiomyopathy Patients: The ESTIMATED Score. *Can J Cardiol* 2020; **36**: 1121-1129.

[28] Lota AS, Tsao A, Owen R, Halliday BP, Auger D, Vassiliou VS, et al. Prognostic Significance of Nonischemic Myocardial Fibrosis in Patients With Normal LV Volumes and Ejection-Fraction. *JACC Cardiovasc Imaging* 2021; **14**: 2353-2365.

[29] Mikami Y, Cornhill A, Heydari B, Joncas SX, Almehmadi F, Zahrani M, et al. Objective criteria for septal fibrosis in non-ischemic dilated cardiomyopathy: validation for the prediction of future cardiovascular events. *J Cardiovasc Magn Reson* 2016; **18**: 82.

[30] Mirelis JG, Escobar-Lopez L, Ochoa JP, Espinosa MA, Villacorta E, Navarro M, et al. Combination of late gadolinium enhancement and genotype improves prediction of prognosis in non-ischaemic dilated cardiomyopathy. *Eur J Heart Fail* 2022; **24**: 1183-1196.

[31] Muthalaly RG, Kwong RY, John RM, van der Geest RJ, Tao Q, Schaeffer B, et al. Left Ventricular Entropy Is a Novel Predictor of Arrhythmic Events in Patients With Dilated Cardiomyopathy Receiving Defibrillators for Primary Prevention. *JACC Cardiovasc Imaging* 2019; **12**: 1177-1184.

[32] Nakamori S, Ngo LH, Rodriguez J, Neisius U, Manning WJ, Nezafat R. T1 Mapping Tissue Heterogeneity Provides Improved Risk Stratification for ICDs Without Needing Gadolinium in Patients With Dilated Cardiomyopathy. *JACC Cardiovasc Imaging* 2020; **13**: 1917-1930.

[33] Neilan TG, Coelho-Filho OR, Danik SB, Shah RV, Dodson JA, Verdini DJ, et al. CMR quantification of myocardial scar provides additive prognostic information in nonischemic cardiomyopathy. *JACC Cardiovasc Imaging* 2013; **6**: 944-954.

[34] Park J, Lee HJ, Kim SK, Yi JE, Shin DG, Lee JM, et al. Smoking aggravates ventricular arrhythmic events in non-ischemic dilated cardiomyopathy associated with a late gadolinium enhancement in cardiac MRI. *Sci Rep* 2018; **8**: 15609.

[35] Perazzolo Marra M, De Lazzari M, Zorzi A, Migliore F, Zilio F, Calore C, et al. Impact of the presence and amount of myocardial fibrosis by cardiac magnetic resonance on arrhythmic outcome and sudden cardiac death in nonischemic dilated cardiomyopathy. *Heart Rhythm* 2014; **11**: 856-863.

[36] Piers SR, Androulakis AF, Yim KS, van Rein N, Venlet J, Kapel GF, et al. Nonsustained Ventricular Tachycardia Is Independently Associated With Sustained Ventricular Arrhythmias in Nonischemic Dilated Cardiomyopathy. *Circ Arrhythm Electrophysiol* 2022; **15**: e009979.

[37] Piers SR, Everaerts K, van der Geest RJ, Hazebroek MR, Siebelink HM, Pison LA, et al. Myocardial scar predicts monomorphic ventricular tachycardia but not polymorphic ventricular tachycardia or ventricular fibrillation in nonischemic dilated cardiomyopathy. *Heart Rhythm* 2015; **12**: 2106-2114.

[38] Purmah Y, Cornhill A, Lei LY, Dykstra S, Mikami Y, Satriano A, et al. Mid-wall striae fibrosis predicts heart failure admission, composite heart failure events, and life-threatening arrhythmias in dilated cardiomyopathy. *Sci Rep* 2022; **12**: 1739.

[39] Shin DG, Lee HJ, Park J, Uhm JS, Pak HN, Lee MH, et al. Pattern of late gadolinium enhancement predicts arrhythmic events in patients with non-ischemic cardiomyopathy. *Int J Cardiol* 2016; **222**: 9-15.

[40] Wu KC, Weiss RG, Thiemann DR, Kitagawa K, Schmidt A, Dalal D, et al. Late gadolinium enhancement by cardiovascular magnetic resonance heralds an adverse prognosis in nonischemic cardiomyopathy. *J Am Coll Cardiol* 2008; **51**: 2414-2421.

[41] Yamada T, Hirashiki A, Okumura T, Adachi S, Shimazu S, Shimizu S, et al. Prognostic impact of combined late gadolinium enhancement on cardiovascular magnetic resonance and peak oxygen consumption in ambulatory patients with nonischemic dilated cardiomyopathy. *J Card Fail* 2014; **20**: 825-832.

# Table S1 – Quality assessment of included studies using the Newcastle-Ottawa scale

| Study | Newcastle-Ottawa Scale | | | |
| --- | --- | --- | --- | --- |
|  | Selection | Comparability | Outcome | Total score |
| LVEF at inclusion ≤35% | | | | |
| Becker 2023 | ★★★ | ★★ | ★★★ | 8 |
| Claver 2022 | ★★★★ |  | ★★★ | 7 |
| Mirelis 2022 | ★★★ | ★★ | ★★ | 7 |
| Piers 2022 | ★★★ |  | ★★★ | 6 |
| Chen 2021 | ★★★★ | ★ | ★ | 6 |
| Infante 2021 | ★★★ | ★ | ★★★ | 7 |
| Alba 2020 | ★★★ | ★★ | ★★★ | 8 |
| Barison 2020 | ★★★★ | ★ | ★★ | 7 |
| Behera 2020 | ★★★ | ★★ | ★★★ | 8 |
| Elming 2020 | ★★★★ | ★★ | ★★★ | 9 |
| Li 2020 | ★★★★ | ★★ | ★★★ | 9 |
| Muthalaly 2019 | ★★★★ | ★★ | ★★ | 8 |
| Park 2018 | ★★★ | ★★ | ★★ | 7 |
| Chimura 2017 | ★★★ | ★★ | ★★★ | 8 |
| Mikami 2016 | ★★★★ | ★★ | ★★ | 8 |
| Shin 2016 | ★★★ | ★★ | ★★ | 7 |
| Chen 2015 | ★★★ | ★ | ★★ | 6 |
| Chimura 2015 | ★★★★ |  | ★★★ | 7 |
| Piers 2015 | ★★★ |  | ★★★ | 6 |
| Perazzolo Marra 2014 | ★★★ |  | ★★★ | 6 |
| Yamada 2014 | ★★★ | ★★ | ★★★ | 8 |
| Neilan 2013 | ★★★★ | ★★ | ★★★ | 9 |
| Gao 2012 | ★★★ |  | ★★ | 5 |
| Leyva 2012 | ★★★★ | ★★ | ★★ | 8 |
| Wu 2008 | ★★★★ | ★★ | ★★ | 8 |
| LVEF at inclusion >35 and <60% | | | | |
| Castrichini 2024 | ★★★ | ★★ | ★ | 6 |
| Hammersley EHJHF 2024 | ★★★ | ★ | ★★ | 6 |
| Hammersley JACC 2024 | ★★★ | ★ | ★★★ | 7 |
| Li 2023 | ★★★ | ★★ | ★★★ | 8 |
| Balaban 2022 | ★★★ | ★★ | ★★ | 7 |
| DeAngelis 2022 | ★★★★ | ★★ | ★★★ | 9 |
| DiMarco 2022 | ★★★★ | ★★ | ★★ | 8 |
| Purmah 2022 | ★★★ | ★★ | ★★★ | 8 |
| Balaban 2021 | ★★★ | ★★ | ★★ | 7 |
| Nakamori 2020 | ★★★★ |  | ★★★ | 7 |
| Halliday 2019 | ★★★ | ★★ | ★★★ | 8 |
| Claridge 2017 | ★★ | ★★ | ★★ | 6 |
| Gulati 2013 | ★★★★ | ★★ | ★★★ | 9 |
| Assomull 2006 | ★★★ | ★★ | ★★★ | 8 |
| LVEF at inclusion ≥60% | | | | |
| Gil 2023 | ★★★ | ★★ | ★★ | 7 |
| Lota 2021 | ★★★★ | ★★ | ★★★ | 9 |
